# Predictive factors for open reduction of flexion-type supracondylar fracture of humerus in children

**DOI:** 10.1186/s12891-022-05798-5

**Published:** 2022-09-14

**Authors:** Jun Sun, Jing Shan, Lian Meng, Tianjing Liu, Enbo Wang, Guoqiang Jia

**Affiliations:** 1 Children’s Hospital of Fudan University Anhui Hospital, Hefei, China; 2 Department of Pediatric Orthopaedics, Shengjing Hospital of China Medical University, Shenyang, China; 3 Anhui Institute of Pediatric Research, Hefei, China

**Keywords:** Humerus, Supracondylar fracture, Flexion, Closed reduction, Risk factors

## Abstract

**Objective:**

The incidence of open reduction and internal fixation (ORIF) in flexion-type supracondylar humerus fractures (SCHF) in children is significantly higher than that of extension-type fractures. This study aimed to identify risk factors for ORIF in flexion-type SCHF.

**Methods:**

One hundred seventy-one patients with Wilkins type III flexion-type SCHF from January 2012 to December 2021 were retrospectively enrolled in a tertiary paediatric hospital. Patients were divided into ORIF group versus closed reduction and internal fixation (CRIF) group. Then, patients data of age, sex, injury side, obesity, deviation of displacement, fracture level, rotation, nerve injury, and delay from injury to surgery were reviewed. Univariate analysis and multivariate logistic regression were used to identify independent risk factors and odds ratios (OR) of ORIF.

**Results:**

Overall, 171 children with type III flexion-type SCHF were analyzed (average aged 7.9 ± 2.8 years). Displacement was lateral in 151 cases, medial in 20. 20 cases had combined ulnar nerve injury. The failed closed reduction rate was 20%. Univariate analysis indicated age, distal fracture fragment rotation, and ulnar nerve injury were significantly associated with ORIF. (*P* = 0.047, *P* = 0.009, and *P* = 0.001, respectively). Multivariate logistic regression analysis showed that distal fracture fragment rotation (OR, 3.3; 95%CI:1.1–9.5; *P* = 0.028) and ulnar nerve injury (OR, 6.4; 95%CI:2.3–18.3; *P* = 0.001) were independent risk factors; however, the age was not an independent one (OR, 1.5; 95%CI:0.6–3.5; *P* = 0.397) for ORIF in the Wilkins type III flexion-type SCHF.

**Conclusion:**

Distal fracture fragment malrotation on initial x-rays and ulnar nerve injury were significant risk factors for ORIF in Wilkins type III flexion-type SCHF. Surgeons should prepare tourniquets or other open reduction instruments when treating these types of fractures.

**Level of evidence:**

Level IV

**Supplementary Information:**

The online version contains supplementary material available at 10.1186/s12891-022-05798-5.

## Introduction

Flexion-type supracondylar humerus fractures (SCHF) in children are rare in clinical practice, accounting for 2–4% of all SCHF [1–3]. The classification of flexion-type SCHF proposed by Wilkins in 1990 defines the degree of displacement as: Type I, minimally displaced with both anterior and posterior cortex integrity; Type II, simple anterior displacement with some anterior cortex integrity; and Type III, displaced without cortex integrity [4]. Treatment algorithm for flexion-type SCHF is similar to that of extension-type SCHF: closed reduction is attempted firstly, followed by open reduction in cases of failed closed reduction [5, 6]. The definition of failure of closed reduction and internal fixation (CRIF) includes mal-alignment radiographic parameters, such as the Baumann angle out of range (64–81) degree and the humerocondylar angle out of (35–45) degree, and the anterior humeral line does not pass through the middle or posterior third of the humerus capitellum [7–9].

The classical techniques for flexion-type SCHF includes 'joystick' and 'push–pull' [10–12]. However, CRIF often fails in flexion-type, and the associated risk factors are unclear [13–15]. There is few relevant research conducted to evaluate the independent risk factors for ORIF of flexion-type SCHF. Furthermore, the independent predictors, used to customize the surgical management for limiting the risk of ORIF individually, still remain unclear. Thus, this study aimed to retrospectively analyze medical records to identify independent risk factors for ORIF in patients with severe displacement Wilkins type III flexion-type SCHF.

## Methods

### Subjects

Data from patients with SCHF aged 1–16 years treated in one tertiary, paediatric, and academic hospital in Anhui province, which is located in Eastern China with a 62-million population, from January 2012 to December 2021 were retrospectively collected. In total, 4301 cases, including 185 flexion-type cases, were recorded.

The inclusion criteria were (1) Wilkins type III fractures; (2) original preoperative anteroposterior and lateral radiographs (with/without preoperative 3D CT); and (3) complete medical records, including height, weight, and operation records. The exclusion criteria were (1) open fracture or vascular injury requiring exploration; (2) manipulation before the original anteroposterior and lateral X-ray films; and (3) combination with fractures in other parts of the ipsilateral limb such as humeral shaft fracture, radial neck fracture, and olecranon fracture.

Overall, 171 patients with an average of 7.86 ± 2.82 years (range 2–15 years) were analyzed. For all patients, CRIF was the first treatment choice, then ORIF was performed if the closed reduction failed. All the procedures were performed by fellowship level surgeons.

### Classification of groups

**Fig. 1:**
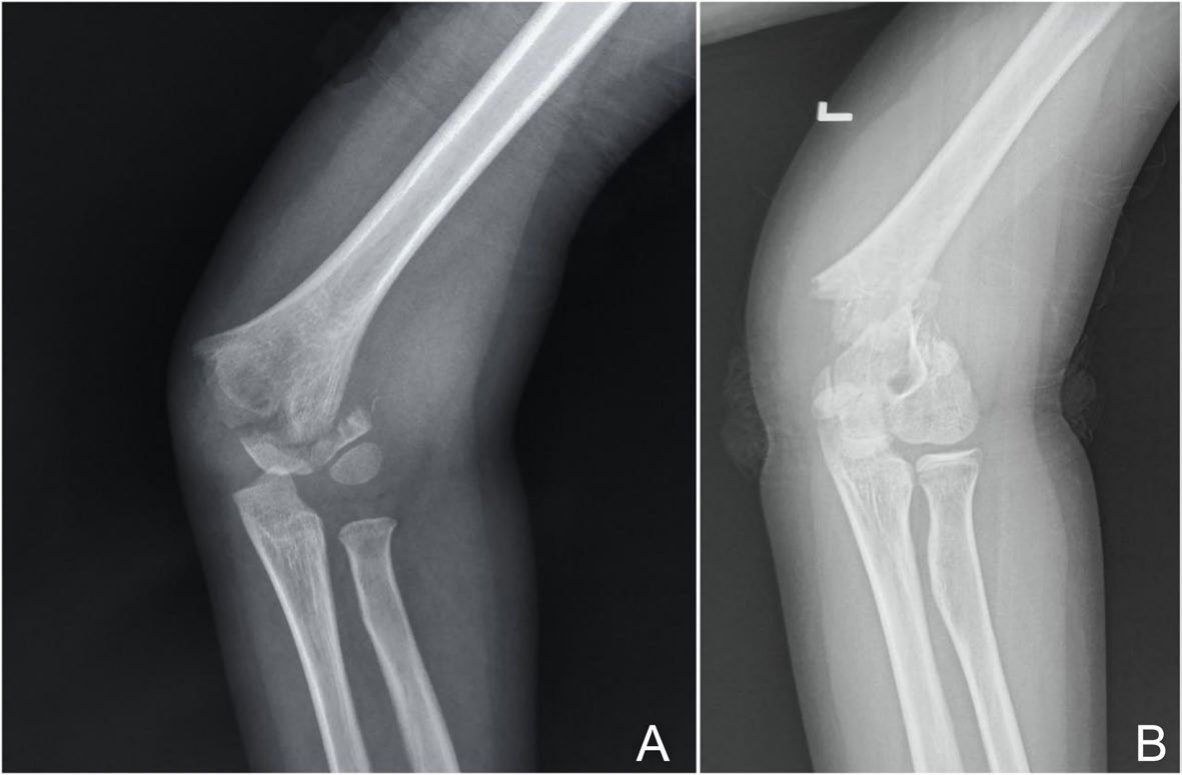
**A** In the anteroposterior view, the fracture level below the isthmus of the distal humerus defined a low-level fracture; and **B** the fracture level above the isthmus of the distal humerus defined a high-level fracture

**Fig. 2:**
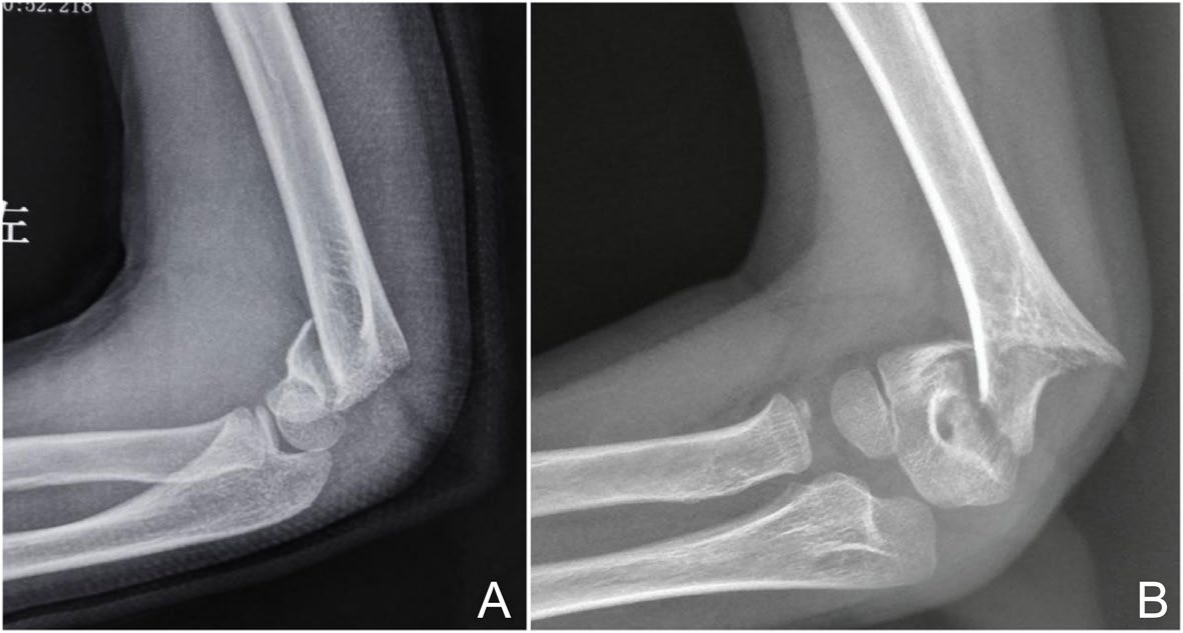
**A** In the lateral view, the distal fragment combined with minimal rotation; **B** In the lateral view, the distal fragment combined with obvious rotation

Patient data included age, sex, injury side, obesity, fracture deviation, fracture level, rotation, ulnar nerve injury, and delay time from injury to surgery. Based on surgical records, patients were divided into ORIF group and CRIF group. The definition of fracture level was the anatomical fracture line below (the low) or above the isthmus of the distal humerus (the high). Patients were divided into two groups (Fig. 1) [16]; age ≥ 10 years represented the adolescent group, and < 10 years were assigned to the child group; patients were classified into four body mass index (BMI) percentile classes: underweight (< 5th percentile), normal (5th–85th percentile), overweight (> 85th–95th percentile), and obese (> 95th percentile). Patients were stratified into medial or lateral deviation based on the displacement direction of the distal fragment. The condition of the distal fracture fragment with/without rotational displacement referent to the longitudinal axis of the proximal humerus, was used to divide patients into the rotation and non-rotation groups (Fig. 2). Patients were also stratified according to presence/absence of ulnar nerve injury, while the time from injury to operation divided patients into < 36 h and ≥ 36 h groups. Data relative to the CRIF and ORIF groups were shown in Table 1.

**Table 1:** Univariate analysis of risk factors for the failure of closed reduction

	ORIF (35)	CRIF (136)	*χ*^*2*^	*P*
Sex			0.622	0.430
Female	17	56		
Male	18	80		
Injury side			0.267	0.606
Left	14	48		
Right	21	88		
Age			3.974	0.047†
≥ 10 yrs	15	35		
< 10 yrs	20	101		
Obesity			0.785	0.376
Yes	7	19		
No	28	117		
Delayed time to operation			1.943	0.163
≥ 36 hours	19	56		
< 36 hours	16	80		
Fracture deviation			3.329	0.068
Medial	1	19		
Lateral	34	117		
Ulnar never injury			21.744	0.001†
Yes	13	7		
No	22	129		
Fracture level			2.030	0.154
High	28	92		
Low	7	44		
Rotation			6.811	0.009†
Yes	30	85		
No	5	51		

^†^ Significant difference, *ORIF* Indicates open reduction and internal fixation, *CRIF* Indicates closed reduction and internal fixation

### Statistical analysis

Descriptive statistics and rates were used to describe the population using means, SD, and percentages. Statistical analysis was conducted using SPSS (version 24.0; IBM, Armonk, New York, United States). The chi-square test or Fisher’s exact test was used to identify associations between different factors and ORIF. A multivariate logistic regression model was used to define the odds of ORIF for each identified risk factor in the univariate analyses. The odds ratios (OR) along with 95% confidence intervals (95% CIs) and respective *P*-values, were calculated. Statistical significance was set at *P* < 0.05.

## Results

Thirty-five cases (20.4%) belonged to the ORIF group and 136 to the CRIF group. There were 151 cases (88.3%) of lateral deviation and 20 cases of medial deviation; 120 cases (71.2%) were high-level fractures and 51 cases were low-level fractures; 20 cases (11.6%) showed combined ulnar nerve injury and 115 cases (67.3%) were in the rotation group.

Univariate analysis revealed sex, injury side, obesity, fracture level, delayed time to operation, and deviation of displacement did not significantly differ between the two groups (*P* > 0.05) (Table 1). Ulnar nerve injury and distal fracture fragment rotation were significant different between the two groups (*P* < 0.05).

Three variables in the univariate analysis satisfied the criteria for inclusion in the multivariate logistic regression model (Table 2). In the multivariate model, two factors were identified as indicators for risk of ORIF. The predicted odds of failure were 3.3 fold higher for the rotation of the distal fragment compared with that of non-rotation group. Furthermore, the OR of ORIF was estimated to be 6.4 fold higher for a patient with ulnar nerve injury.

**Table 2:** Multivariate analysis of risk factors for the ORIF

	ORIF		
Risk Factors	OR	95% CI	*P*
Age	1.5	0.6–3.5	0.397
Direction of displacement	4.7	0.6–39.6	0.156
Rotation	3.3	1.1–9.5	0.028†
Ulnar never injury	6.4	2.3–18.3	0.001†

^†^ Significant difference, *OR* Odds ratio, *CI* Confidence interval

## Discussion

The ORIF rate in flexion-type SCHF was significantly higher than the classic extension-type [10–12]. In previous studies from other hospitals, the ORIF rate of flexion-type SCHF ranged from 14 to 28% [10–15]. In this study, the overall closed reduction failure rate was 20%. The failure of closed reduction was combined with some risk factors.

This study retrospectively analyzed the data of children with Wilkins type III flexion-type SCHF in our hospital over the past 10 years, and drew meaningful conclusions for surgeons. The ulnar nerve injury and rotation of the distal humeral fragment were identified as two independent risk factors, which could be used to customize the surgical management for limiting the risk of failure on individual.

Flexion-type SCHF are prone to damage the ulnar nerve, and the ORIF rate is significantly higher [10, 13, 17–19]. When the distal fracture fragment had severe rotation displacement, the patient showed subcutaneous ecchymoses on the posterior-medial side of the elbow joint, often accompanied by a medial metaphysis spike of the proximal humerus on the radiograph. The rotated medial spike may injure the ulnar nerve, puncture the nerve sheath, or increase the risk of ulnar nerve entrapment during closed reduction. In addition, the spread of the periosteum and the penetration of the triceps muscle of the medial spike increases the difficulty of CRIF [20, 21]. Furthermore, the medial periosteum disruption aggravates instability and the medial column rotational displacement always occurs during closed reduction. Intraoperative maintenance of medial column stability is the key to CRIF. Although some patients showed stable fractures on initial X-ray films, the fractures were actually found to be unstable during the operation due to the complete disruption of the medial periosteum. Altogether, this results in a significant increase in the ORIF rate.

Previous studies of Gartland extension-type III supracondylar humerus fractures, confirmed that fracture type, injury mechanism, time from injury to operation, medial spike, obesity, and lateral deviation were risk factors for ORIF, and showed that flexion-type was much tougher to maintain closed reduction [22–30]. In our study, indicators such as obesity, time from injury to operation, displacement deviation, and fracture level were tested as binary variables, none of which influenced the treatment plans. Obesity significantly increases the difficulty of closed reduction in the extension type, which is different from the results of our study in the flexion type [23, 24]. Although the results showed no significance difference between lateral and medial displacement of flexion-type SCHF, there is a tendency and our personal opinion and experience suggests that is more difficult to achieve CRIF. Although the lateral side is more stable during the reduction process, it often combines with irreducible medial side rotation and medial column periosteum disruption, leading to the failure of closed reduction [25]. Regarding to the fracture level, the high-level fracture is more unstable because the proximal bone contact area above the olecranon fossa isthmus is smaller than the distal part, so it is commonly difficult to be succeeded in CRIF. However, our findings indicated that the fracture level did not influence the treatment method [30].

It is difficult for older children to achieve a successful CRIF in clinical practice, which may be associated with the stronger muscles of the patients. In our study, the age was a risk factor for ORIF by univariate analysis; however, there were no significant difference when analysed by multivariate regression in this study. Similarly, older age did not increase the risk of ORIF by a study of Bekmez [26]. Different studies have drawn conflicting conclusions regarding delayed time-to-operation. One indicated that delayed treatment after 24 h will lead to an increased probability of ORIF [27, 28]. Instead, Yang et al. using the same ethnic origin population as ours also confirmed that delayed treatment for 8 h to 5 days increased the ORIF rate [29]. Conversely, delayed treatment may not influence the risk of ORIF and emergency surgery at night is not recommended [31, 32]. The delayed time to operation was defined as 36 h as many patients spend over 24 h when they came into the emergency room in developing countries, and additional hours for preoperative preparation should be considered. Our findings suggested that a delay ranging from 36 h to 7 days did not increase the risk of ORIF.

In this study, there are some limitations. Firstly, as a retrospective study, the surgical records may not have been sufficiently detailed, which may have caused information bias. Secondly, only 1/10 medial deviations in displacement patients were included in this study; therefore, the result of deviation may come with a bias. Thirdly, all the surgeons were at fellowship level that it may present a bias on experience. Finally, we converted continuous variables into categorical variables, which may have affected the results.

In conclusion, combined ulnar nerve injury and malrotation on initial x-rays of the distal fragment were two risk factors for ORIF in flexed Wilkins type III supracondylar humerus fractures. These new findings will facilitate preoperative decision-making by surgeons.

## Supplementary Information

**Additional file 1.**

## Data Availability

All data generated or analysed during this study are included in this published article [and its supplementary information files].

## References

[1] Cheng JC, Lam TP, Mafulli N (2001). Epidemiological features of supracondylar fractures of the humerus in Chinese children. J Pediatr Orthop B.

[2] De Boeck H (2001). Flexion-type supracondylar elbow fractures in children. Journal of Pediatric Orthopaedics.

[3] Zorrilla SNJ, Prada-Cañizares A, Marti-Ciruelos R (2015). Supracondylar humeral fractures in children: current concepts for management and prognosis. Int Orthop.

[4] Wilkins KE (1990). The operative management of supracondylar fractures. Orthop Clin North Am.

[5] Mulpuri K, Hosalkar H, Howard A (2012). AAOS clinical practice guideline: the treatment of pediatric supracondylar humerus fractures. J Am Acad Orthop Surg.

[6] Garg B, Pankaj A, Malhotra R (2007). Treatment of flexion-type supracondylar humeral fracture in children. J Orthop Surg (Hong Kong).

[7] Ojeaga P, Wyatt CW, Wilson P (2020). Pediatric type II supracondylar humerus fractures: factors associated with successful closed reduction and immobilization. J Pediatr Orthop.

[8] Madjar-Simic I, Talic-Tanovic A, Hadziahmetovic Z (2012). Radiographic assessment in the treatment of supracondylar humerus fractures in children. Acta Inform Med.

[9] Kao HK, Lee WC, Yang WE (2016). Clinical significance of anterior humeral line in supracondylar humeral fractures in children. Injury.

[10] Flynn K, Shah AS, Brusalis CM (2017). Flexion-type supracondylar humeral fractures: ulnar nerve injury increases risk of open reduction. J Bone Joint Surg Am.

[11] Kuoppala E, Parviainen R, Pokka T (2016). Low incidence of flexion-type supracondylar humerus fractures but high rate of complications. Acta Orthop.

[12] Steinman S, Bastrom TP, Newton PO (2007). Beware of ulnar nerve entrapment in flexion-type supracondylar humerus fractures. J Child Orthop.

[13] Chukwunyerenwa C, Orlik B, El-Hawary R (2016). Treatment of flexion-type supracondylar fractures in children: the ‘push–pull’ method for closed reduction and percutaneous K-wire fixation. J Pediatr Orthop B.

[14] Green BM, Stone JD, Bruce RW (2017). The use of a transolecranon pin in the treatment of pediatric flexion-type supracondylar humerus fractures. J Pediatr Orthop.

[15] Kao HK, Lee WC, Yang WE (2017). Treatment of displaced flexion-type pediatric supracondylar humeral fractures in the prone position. J Orthop Surg (Hong Kong).

[16] Kang S, Kam M, Miraj F (2015). The prognostic value of the fracture level in the treatment of Gartland type III supracondylar humeral fracture in children. Bone Joint J.

[17] Kim KY, Conaway W, Schell R (2020). Prevalence of ulnar nerve palsy with flexion-type supracondylar fractures of the humerus. J Pediatr Orthop B.

[18] Turgut A, Kalenderer Ö, Bozoğlan M (2015). Flexion type supracondylar humerus fractures: 12 year experience of a pediatric orthopedics clinic. Eklem Hastalik Cerrahisi.

[19] Delniotis I, Dionellis P, Gekas CC (2020). Flexion-type supracondylar humeral fracture with ulnar nerve injury in children: two case reports and review of the literature. Am J Case Rep.

[20] Schultz JD, Rees AB, Wollenman LC (2022). Bruise location in supracondylar humerus fractures predicts specific neurovascular injuries. J Pediatr Orthop.

[21] Lim KB, Lim CT, Tawng DK (2013). Supracondylar humeral fractures in children: beware the medial spike. Bone Joint J.

[22] Novais EN, Carry PM, Mark BJ (2016). Posterolaterally displaced and flexion-type supracondylar fractures are associated with a higher risk of open reduction. J Pediatr Orthop B.

[23] Çabuk H, Dedeoğlu SS, Adaş M (2016). Medial spike and obesity associate with open reduction in type III supracondylar humeral fracture. Acta Chir Orthop Traumatol Cech.

[24] Li NY, Bruce WJ, Joyce C (2018). Obesity’s influence on operative management of pediatric supracondylar humerus fractures. J Pediatr Orthop.

[25] Prusick VW, Gibian JT, Ross KE (2021). Surgical technique: closed reduction and percutaneous pinning of posterolaterally displaced supracondylar humerus fractures. J Orthop Trauma.

[26] Bekmez S, Camp MW, Ling R (2021). Supracondylar humerus fractures in older children: success of closed reduction and percutaneous pinning. J Pediatr Orthop.

[27] Walmsley PJ, Kelly MB, Robb JE (2006). Delay increases the need for open reduction of type-III supracondylar fractures of the humerus. J Bone Joint Surg Br.

[28] Okkaoglu MC, Ozdemir FE, Ozdemir E (2021). Is there an optimal timing for surgical treatment of pediatric supracondylar humerus fractures in the first 24 hours?. J Orthop Surg Res.

[29] Yang J, Sun LJ, Du Shenghu (2013). Risk factors of failure in closed reduction for supracondylar fractures of humerus in children. Chin J Pediat Surg.

[30] Beck JD, Riehl JT, Moore BE (2012). Risk factors for failed closed reduction of pediatric supracondylar humerus fractures. Orthopedics.

[31] Sibinski M, Sharma H, Bennet GC (2006). Early versus delayed treatment of extension type-3 supracondylar fractures of the humerus in children. J Bone Joint Surg Br.

[32] Abdelmalek A, Towner M, Clarke A. Are we staying up too late? Timing of surgery of displaced supracondylar fractures in children. Clinical audit in a paediatric tertiary UK trauma centre and literature review. Arch Orthop Trauma Surg. 2022;9. 10.1007/s00402-021-04289-x. 10.1007/s00402-021-04289-x 34999994

