# Supplementary material for: Predictive factors for open reduction of flexion-type supracondylar fracture of humerus in children
Source: BMC Musculoskelet Disord. 2022 Sep 14;23:859. doi: 10.1186/s12891-022-05798-5 (PMC9472328; doi:10.1186/s12891-022-05798-5)
Supplement: Supplementary file 1 — Additional file 1. [file 12891_2022_5798_MOESM1_ESM.docx]

“Availability of Data and Materials” statements

The datasets generated and/or analysed during the current study are not publicly available due to further research on this type fracture patients but are available from the corresponding author on reasonable request.
